# Cases of Disease of the Eye from Affections of the Teeth

**Published:** 1882-04

**Authors:** Edward T. Ely


### ILLUSTRATIVE CASES OF DISEASE OF THE EYE ARISING FROM AFFECTIONS OF THE TEETH.

It is a familiar fact that serious disease of the eye may be caused through affections of the fifth nerve in its distribution to distant parts. The following four cases (from the practice of Dr. Roosa and myself) are offered in illustration of the subject, because they were very carefully observed, and because in each one the relation of cause and effect seems unmistakable. In all the affection of the eye appeared to arise from irritation about the teeth.

CASE I.—*Paresis of Orbicularis Muscle—Irregular Spasm of Ciliary Muscle—Monocular Diplopia.*—Male, aged twenty-six. Complains that vision of right eye has suddenly become blurred, and that he sees double with that eye. No pain or redness. Pupil small and movable. Fundus normal. Has paresis of right orbicularis ; lids cannot be completely closed, and eye is very watery. V. $=\frac{20}{50}$ and with $+\frac{1}{36}$ c. 180° $=\frac{20}{20}$. A careful examination of the teeth shows nothing abnormal.

Patient was ordered to take mercury and iodide of potash, which he did for some time without benefit. One night he was seized with severe pain in one of his upper molar teeth. The next day the tooth was extracted, and an abscess which had formed about its root was evacuated. Paresis of orbicularis muscle, diplopia and astigmatism disappeared immediately, and V. became $\frac{20}{20}$ without any glass. There was no doubt about the astigmatism in this case, as the vision was subjected to the most careful tests.

CASE II.—*Paresis of Right Internal Rectus and Ciliary Muscles.*— Male, aged thirty-one. December 15, 1880, complains of blurring and "confusion" of vision of right eye of a week's duration. No redness or pain. Size and movements of pupil normal. Fundus normal. V. $=\frac{20}{20}$, with $+\frac{1}{60}$. Slight paresis of right internal rectus muscle. Slight paresis of accommodation, requiring $+\frac{1}{16}$ to restore normal range.

Root of first molar tooth of upper jaw, right side, is denuded, roughened, and sensitive. Patient was referred to a dentist, and was also treated with mercury and iodide of potash (there being a syphilitic history), and by electricity. Part of the root of the tooth was removed and the remainder filled. The nerve of the tooth was found "dead," and the alveolar process absorbed, but there was extensive suppuration in the adjacent parts. The ocular paresis recovered immediately when the condition of the tooth was corrected (in the latter part of January), the other treatment having been abandoned for some time previously.

CASE III.—*Partial Paresis of Third Nerve.*—Female, aged forty. June 3, 1881, complains of confused feeling in right eye, which she cannot describe. Says it began with burning pain in the right ear and the right side of the head.

No redness of eye. Pupil dilated and immovable, and accommodation partially paralyzed. Opacities of both lenses. Teeth on right side are decayed and tender, and gums are in an unhealthful condition. Advised to consult a dentist.

June 9th.—Paresis of third nerve disappeared entirely after extraction of one tooth.

Case IV.—*Inflammation of Conjunctiva and Sclera* (?).—Male, aged thirty-three. January 20, 1882, complains of painful inflammation of left eye, which he has had for three weeks. Has had " neuralgia" of left side of face, most of the time, for the past month.

There is a patch of inflammation, involving conjunctiva, subconjunctival tissue, and apparently the sclerotic, at the lower and outer quadrant of the globe. It is about ten millimetres broad, and extends from the edge of the cornea to the retrotarsal fold. Its appearances are those of the affection ordinarily called episcleritis. There is lachrymation and localized ciliary tenderness. The pupil is small, but movable. Vision and accommodation normal. Fundus normal. Between first and second canine teeth of upper jaw, left side, is a small, ulcerated patch on the gum. Space between the teeth is very tender upon pressure with probe.

Patient was advised to consult a dentist, who found an exposed nerve in one of the canine teeth just referred to. An application was made to devitalize the nerve, and the facial neuralgia disappeared at once. The ocular inflammation disappeared completely within forty-eight hours. No local treatment for the eye was employed.—*Dr. Edward T. Ely, in Medical Record.*

